# Lay worker-administered behavioral treatments for psychological distress in resource-limited settings: Time to move from evidence to practice?

**DOI:** 10.1371/journal.pmed.1002372

**Published:** 2017-08-15

**Authors:** Alexander C. Tsai

**Affiliations:** 1 Chester M. Pierce, MD Division of Global Psychiatry, Massachusetts General Hospital, Boston, Massachusetts, United States of America; 2 Harvard Medical School, Boston, Massachusetts, United States of America; 3 Mbarara University of Science and Technology, Mbarara, Uganda; 4 MGH Global Health, Massachusetts General Hospital, Boston, Massachusetts, United States of America

## Abstract

In this Perspective on the study by Bryant and colleagues, Alex Tsai discusses the evidence supporting lay worker-administered, behavioral interventions for women survivors of interpersonal violence.

Exposure to nonlethal violence is, apart from its resulting physical injury or other adverse health sequelae, one of the most consistently estimated causal risk factors for psychological distress, mental and substance use disorders, suicidal behaviors, and completed suicide [1–7]. Throughout sub-Saharan Africa, a strong gendered patterning in these exposures has been observed, and the rates of intimate partner and nonpartner violence against women in this region are among the highest in the world [8–11]. Even were health systems in sub-Saharan Africa equipped, contrary to fact [12, 13], to handle the additional burden of mental health service delivery for affected populations, most survivors of violence never seek or obtain proper treatment [8]. In many settings, care-seeking behaviors are severely compromised by stigma, gender-inequitable norms, and inadequate legal protections [14]. Further, screening of asymptomatic women has not been shown to be a useful approach for improving health outcomes [15, 16].

In the face of these significant structural challenges, considerable progress has been made in the past 2 decades on the development, implementation, and evaluation of behavioral interventions in resource-limited settings. Recently published studies have convincingly demonstrated that lay health workers can be trained and supervised to deliver low-intensity behavioral interventions (“task shifting”) of adequate dose and timing to improve health outcomes in a variety of vulnerable populations, including pregnant women [17–21]; adults with common mental disorders [22–25], schizophrenia [26], and alcohol use disorders [27]; and survivors of torture and other forms of interpersonal violence [28–30]. Thus, task shifting should be viewed as a valid and effective approach for expanding access to evidence-based psychological interventions in settings where underfunded mental health systems lack the capacity to address unmet population mental health needs.

It is in this context that we celebrate the publication, in this issue of *PLOS Medicine*, of the findings from a randomized controlled trial by Bryant and colleagues [31] designed to estimate the efficacy of a brief, manualized, lay worker-administered, behavioral intervention (“Problem Management Plus,” or PM+) on reducing psychological distress among women survivors of interpersonal violence living in periurban settlements near Nairobi, Kenya. Of note, the PM+ intervention tested in this study—and in a companion study conducted in Pakistan, the findings of which were recently published by Rahman and colleagues [32]—is of lower intensity compared with most of the interventions tested in the studies cited above. Bolton and colleagues [22], for example, tested the efficacy of a 16-session group interpersonal therapy intervention, while Bass and colleagues [28] tested the efficacy of a 12-session individual and group cognitive processing therapy intervention. PM+ adopts a “transdiagnostic” approach, which presses treatment elements commonly employed in evidence-based psychological interventions (e.g., psychoeducation, behavioral activation, and cognitive restructuring) into the service of addressing symptoms characteristic of a broad range of distress states [33]. These distress states are referred to as “common mental disorders” because they are often encountered in the community or at the level of primary care, frequently manifesting with a mixed picture of anxiety, depressive, and unexplained somatic symptoms. Transdiagnostic interventions are particularly amenable to task shifting to meet the mental health burden occasioned by such presentations, because they skirt the need for lay health workers to formulate a definitive or even working diagnosis and select from a portfolio a theory-driven intervention to address the hypothesized basis for the observed signs and reported symptoms—tasks that may be especially challenging for already overburdened lay health workers when a patient presents with potentially comorbid mental disorders and overlapping symptoms. Thus, while the findings of this study’s predecessors [17–30] all support the feasibility of the task-shifting approach, PM+ may have even greater appeal in settings where implementation and supervisory capacity are particularly limited.

Bryant and colleagues [31] enrolled 421 women with a history of exposure to interpersonal violence and who were impaired by psychological distress. Three-quarters reported a lifetime history of physical violence, and one-half a history of sexual violence; one-fifth reported suicidal ideation in the month prior to enrollment. Remarkably, the study also included, but did not emphasize the data from, 97 women who met the inclusion criteria for impairment and distress but who did not report a history of exposure to interpersonal violence. This design choice permitted the investigators to minimize any potential stigma [14] attached to participation in a study that otherwise would have been framed as targeting survivors of interpersonal violence. After 3 months of follow-up, with a 24% attrition rate, study participants allocated to PM+ had a 3.3 lower mean score on the General Health Questionnaire compared with participants assigned to enhanced usual care (EUC, or nurse-provided, nonspecific counseling). Expressed in terms of its effect size, the magnitude of this observed treatment effect is comparable to what has been observed in short-term randomized trials of antidepressant medication [34, 35] and collaborative care [36, 37] treatment of depressive disorders. Smaller differences were observed on the secondary outcomes of functional impairment and symptoms of post-traumatic stress, perhaps suggesting broad-spectrum, transdiagnostic utility.

Bolstered by the findings of 2 positive studies conducted in very different cultural contexts [31, 32], is PM+ ready for widespread implementation? Our collective interpretation of the estimates presented by Bryant and colleagues [31] is subject to 3 important limitations, 2 of which were touched upon by the authors.

First, while allocation to the PM+ intervention arm appears to have reduced symptom severity across several domains, because of the study design, we lack certainty about whether the observed treatment effects resulted from PM+ itself or from other potentially confounding influences linked to treatment allocation. Uptake of the intervention was relatively high, with more than one-half of women allocated to the PM+ arm ultimately completing all 5 sessions (compared with a much smaller proportion of women allocated to the EUC arm), and the number of sessions attended was correlated with greater symptom reduction. Further, as the authors note, the home visit itself could have acted as a salubrious active ingredient independent of any effects of the PM+ components, given that the EUC sessions took place at primary health care centers and also given that systematic differences in contact time—which was uncontrolled—could have emerged. Neither is there evidence of mechanistic specificity, which could have been provided (e.g., by collecting data on potentially mediating variables such as the Behavioral Activation for Depression-Short Form [38]) had concerns about respondent burden not required the authors to minimize the length of the survey. Therefore, it is possible that the observed treatment effects represent, at least in part, nontrivial biases away from the null.

A second important limitation of the study is the short duration of follow-up. Certainly, the common mental disorders targeted by PM+ and the other brief interventions tested in this literature are characterized by symptoms and impairment of relatively low severity. On the other hand, effective treatment of syndromal illnesses prone to relapse or recurrence, such as major depressive disorder or post-traumatic stress disorder, would require the mobilization of considerably more resources for continuation and maintenance treatment. For example, up to one-half of patients with major depressive disorder experience recurrent episodes in a given year [39–42], and the median duration of episodes is 3–5 months [39, 43, 44]. Presumably, if resources were so limited in a particular setting that a brief behavioral intervention such as PM+ was being considered for widespread implementation, then its use for longer-term maintenance treatment would be out of the question. To demonstrate efficacy in preventing recurrence of future illness episodes, a study would need to show benefit for at least 6 months after remission of symptoms [45, 46]. Alternatively, PM+ could potentially be embedded within a larger collaborative care treatment apparatus, its role limited to episodic, short-term treatment of psychological distress associated with significant impairment (with tailored alternatives such as medication management to be made available for nonresponders or treatment-refractory cases); evidence to support efficacy in this context could be derived from a sequential multiple assignment randomized trial to estimate an optimal dynamic treatment regime.

Third, while Bryant and colleagues [31] elicited women’s histories of exposure to interpersonal violence upon entry into the study, they did not assess re-exposure to interpersonal violence during the study as a secondary outcome. Their failure to do so represents somewhat of a missed opportunity. (It should be noted that statistically significant improvements were not observed on the Life Events Checklist, which includes exposure to physical and sexual violence among the 16 events assessed, but these were not analyzed separately.) In general, there has been little discussion about the potential role for individual-level behavioral interventions in the secondary and tertiary prevention of violence against women [47]. Concerns about victim blaming have hampered empirical research into understanding how individual-level characteristics of survivors may be predictive of subsequent re-exposure to interpersonal violence [5, 48]. Acknowledging the need for this sensitivity, it should be noted that a key role has been proposed for individual-level variables such as psychological distress, relational disruption, self-blame, emotional numbing, impaired risk perception, and affect dysregulation in conceptual models linking exposure and re-exposure to interpersonal violence [49–54]. Consistent with these models, longitudinal studies in multiple contexts have demonstrated a potentially bidirectional relationship between exposure to interpersonal violence and elevated psychological distress [5, 54–58]. Similarly, the United States–based randomized controlled trial by Iverson and colleagues [59] found that a cognitive processing therapy intervention to treat symptoms of depression and post-traumatic stress among women survivors of interpersonal violence successfully reduced the probability of re-exposure to violence at 6-month follow-up. Given these disparate strands of research, it is possible that brief interventions like PM+ might exert similar beneficial preventive impacts on interrupting the vicious cycle between interpersonal violence and psychological distress—perhaps either only in settings where a broad array of related services are available (e.g., case management, safety planning, crisis services, legal advocacy, emergency shelters, transitional housing, and/or parenting and childcare support [60]) or through combination interventions in which some of these elements are packaged together with PM+. At this time, the field will have to wait until the next study before these hypotheses are adequately tested.

Despite these limitations, PM+ has considerable appeal. The nature of the intervention content augurs well for the possibility of finding evidence of longer-term benefit, given that the skills learned during treatment sessions can be practiced long after treatment is discontinued. This feature would give PM+ and related behavioral interventions an advantage over medication-driven treatment strategies [61] in settings where the rare prescriber is available yet pharmacies are beleaguered by frequent stock outs. Even if PM+ turns out not to be cost saving, it may still be cost-effective in providing value for money. One might reasonably expect the costs (i.e., of training, of visits occurring with greater frequency during acute-phase treatment, etc.) to be front-loaded and for any incremental cost-effectiveness ratios to be more favorably estimated in studies of longer duration. Further evidence to support the cost-effectiveness of PM+ might be gained by directly measuring collateral outcomes related to functional impairment of index participants, including improved economic productivity [22, 62–65] or parenting behaviors (and attendant child outcomes) [66, 67], or reduced need for informal support from family caregivers [68].

The potential widespread deployment of PM+ stands at the intersection of 2 vital issues relevant to women’s health: mental health and interpersonal violence. Until the large-scale structural forces that give rise to health disparities affecting vulnerable populations can be eliminated—poverty, gender-inequitable norms, differences in social and economic power, and so forth [60, 69, 70]—the health system will continue to play a key role in the multisectoral response to violence against women in resource-limited settings. Bryant and colleagues [31] appropriately recognize the need for long-term implementation trials to establish the feasibility and sustainability of PM+ when deployed at scale. Their study is a welcome step toward forming an evidence-based foundation for an effective health sector response in this regard. More work is urgently needed.

## Abbreviations

EUC: enhanced usual care
PM+: Problem Management Plus
